# Challenges in HIV prevention and treatment among men who have sex with men in China: structural exclusion, healthcare inequity, and testing avoidance

**DOI:** 10.3389/fpubh.2026.1841965

**Published:** 2026-07-03

**Authors:** Xiaoting Hao, Yongyuan Wang, Cong Zhang, Yongjie Luo

**Affiliations:** 1 Department of Scientific Research, Xiangyang No.1 People’s Hospital, Hubei University of Medicine, Xiangyang, China; 2 School of Journalism and Communication, Beijing Institute of Graphic Communication, Beijing, China; 3 Second Clinical Medical School, Lanzhou University, Lanzhou, China

**Keywords:** AIDS, health inequality, HIV, men who have sex with men, social discrimination

## Abstract

Men who have sex with men (MSM) in China face a high burden of HIV infection. While biomedical interventions are crucial, technology alone cannot explain the challenges of AIDS prevention and treatment in this group; deeper social structural factors need urgent attention. This study analyzes the ways that structural barriers, through the national policy system and structural stigma, affect the HIV prevention and treatment behaviors of MSM in China. We reviewed central-level academic and policy documents and analyzed the data using NVivo 12. We then integrated the research results from these documents using cross-analysis, which revealed three interrelated structural obstacles. First, the conflict between a series of anti-discrimination laws and HIV being a disqualifying condition in the civil service recruitment physical examination has formed an exclusive employment structure. This conflict has normalized employment discrimination and weakened the social and economic foundations to prevent and treat HIV in MSM. Second, unequal medical resources, the cross-stigmatization of HIV-positive status and homosexual orientation, and the lack of legal recognition of same-sex sexual relations have resulted in the refusal of medical and differential treatment in medical institutions. Third, involuntary identity disclosure promotes HIV testing evasion. In social networks with strong rural characteristics in China, the risk of involuntary identity disclosure shifts detection from health-protective behavior to threat perception, amid weakened social support. This study holds that the AIDS epidemic among MSM is a major public health challenge facing China at present, and effective control requires a paradigm shift to promote health equity. We suggest taking advantage of the existing connections among the MSM group on localized social media to promote HIV prevention and health equity in this population.

## 1 Introduction

The human immunodeficiency virus (HIV) remains a major challenge in the field of public health in China. On December 6, 2024, the General Office of the State Council of China issued China’s Plan to Curb and Prevent AIDS (2024–2030), which stressed the need to control the epidemic at a low level and pointed out that sexual contact has become the main vector of AIDS transmission in China (1). While the AIDS epidemic in China is generally at a low prevalence level, the large population base means that the cumulative number of infected people is still large. As of December 31, 2024, there are 749,839 people living with HIV and 605,178 patients with AIDS nationwide (excluding Hong Kong, Macao, and Taiwan), 25,615 of whom contracted the disease through homosexual transmission, accounting for 25.2% (2). In 2001, men who have sex with men (MSM) in China accounted for only 0.2% of the number of people with AIDS (3). The significant growth in infections among MSM represents an increasingly severe barrier to achieving the “Healthy China 2030” strategic goals. Biomedical interventions, including pre-exposure prevention (PrEP), post-exposure prevention (PEP), and antiretroviral therapy (ART), have made significant progress in the prevention and control of HIV in China (4), but the willingness to use them among MSM is still low, which significantly affects HIV detection and treatment in this group (5). Therefore, relying solely on biomedical technology cannot fully address the high-risk status and inactive intervention and treatment behaviors of this population of patients with HIV, and deeper social structural factors urgently need attention.

Structural discrimination refers to the restriction of specific groups’ access to resources, social participation, and the protection of rights through institutional practices, policy formulation, and cultural norms, thereby affecting their health outcomes. The global academic community has focused on the impact of structural factors on the HIV/AIDS epidemic among MSM, including disease stigmatization (6), religious conflicts (7), racial discrimination (8, 9), discrimination based on sexual minority status (10), socioeconomic status discrimination, and immigration status (11, 12). However, structural barriers are highly context-dependent, and China presents a unique configuration. Although homosexuality was removed from the list of mental illnesses in 2001, and the LGBT movement is not legally prohibited at the national level as it is in Russia, the government’s failure to recognize same-sex sexual orientation has prevented Chinese MSM from forming a legally protected minority identity. Mainstream ideology often imposes strict dissemination restrictions on homosexuality-related media content through legal regulations, administrative management, and value guidance of content censorship. This gray area, neither recognition nor conviction, creates a unique structural vulnerability. In this context, the structural factors affecting the HIV epidemic among the Chinese MSM population have also attracted widespread academic attention, producing multi-level research findings spanning macro-ecology and micro-dynamics. Research highlights a lack of policy support (13), exclusionary employment policies (14), and deep-rooted concepts of filial piety within the Confucian cultural context (15). Gong et al.’s bibliometric research on homosexuality in China over the past decade in particular emphasizes the deep level of filial piety as a unique cultural factor that has a key impact on the well-being of Chinese MSM (16), as well as the systemic obstacles posed by social stigma (15, 17, 18) and the uneven allocation of geographical resources (19). Furthermore, demographic characteristics such as socioeconomic status and education level (13, 19), psychological resilience (20, 21), internalized homophobia (22, 23), and rural identity (15) are intertwined and affect individuals’ risk mitigation and medication adherence behaviors.

These studies provide a foundation for understanding the structural barriers to MSM HIV/AIDS prevention and control in the Chinese context. Overall, the research tends to treat policy texts as a backdrop, offering a relatively fragmented analysis of the current situation. Global research on HIV prevention and treatment has gradually shifted from explanations of individual behavior to analyses of structural inequality, emphasizing how the linkages among policy, knowledge, and service accessibility shape risk exposure and prevention opportunities. Evidence regarding the MSM population indicates that access to PrEP, testing, and basic prevention supplies does not depend on individual willingness alone but is jointly constrained by health education resources, medical resource distribution, and stigmatizing environments. Notably, the knowledge gap regarding PrEP is particularly prominent among Chinese MSM (24). A global survey across different populations revealed that MSM generally face significant inequalities with respect to accessibility, even for preventive services that are considered low-cost or basic. The combination of stigma and medical inaccessibility further exacerbates these disparities (25). A cross-population global survey found that, even when barriers are perceived as related to low cost or basic services, such research more convincingly demonstrates that structural exclusion has contextualized generative characteristics, and its manifestations vary with health systems, social norms, and organizational thresholds (25).

In the Chinese context, this issue is not merely about whether individuals are aware of prevention methods, but rather about whether they can safely acquire and use them. A study examining testing among young mobile MSM revealed that structural barriers significantly influence their testing behavior, indicating that service participation is a decision closely tied to social context and social structure (26). A more detailed study focusing on the disclosure of information by individuals infected with HIV reveals that in environments where traditional gender and sexual norms exert strong pressure, the decision to disclose often comes with expectations of discrimination, relationship breakdown, and social consequences. Therefore, identity concealment serves not only as a social adaptation strategy but also as an important mediating condition that affects subsequent interactions related to seeking help, testing, and ongoing treatment (27). From a macro perspective, research by Link and others has focused on the relationship between policy and stigma, revealing that policy can either alleviate stigma or solidify it as a structural constraint through neglect, regulation, and institutional exclusion (28). Drawing on this work, a joint international study developed a framework of health stigma and discrimination that views stigma as a determinant of health and emphasizes its concurrent impact on healthcare-seeking behavior, ongoing care, and treatment implementation (29). Consistent with this, reviews on HIV stigma and treatment have demonstrated a stable association between stigma and Anti-Retroviral therapy (ART) adherence, often manifested through mediating mechanisms such as psychological stress, avoidance of medical care, and service disruptions. Empirical studies on patients with AIDS have also found that higher levels of internalized stigma are significantly associated with poorer healthcare accessibility and are accompanied by outcomes such as insufficient ART adherence and a lack of stable medical resources (30, 31). However, such studies often regard stigma as an external pressure that acts on individuals and seldom delve into how it is re-encoded and continuously amplified by policies, procedures, and strategies at the level of service implementation. An intersectional study pointed out that policies, protocols, and operational arrangements in HIV services may intentionally or unintentionally reproduce discrimination, embedding inequality in daily practices such as screening, referral, confidentiality, and resource allocation (32). The endogenous operation of policies, selective neglect, and the mechanisms of their reproduction within the institutional chain still require further consideration (28).

Therefore, merely summarizing structural issues as general stigmatization is insufficient to explain how they are specifically embedded in policy texts, organizational rules, and service processes, and how they are continuously amplified in daily medical interactions. Relevant research has begun to shift the focus of exclusion from the attitudinal level to the institutional grammar level, highlighting how unequal discourse becomes policy and is encoded in practice. In this context, exclusion no longer manifests as explicit denial but rather transforms into sustainable action constraints through delay, diversion, additional conditions, and implicit screening (33). The significance of this perspective lies in its ability to elevate structural barriers from the level of stigma existence to the operational level of exclusion, indicating that policies are not merely external contexts but continuously generate visible and accessible differences in organizational implementation, thereby shaping individuals’ expectations of the costs of seeking help, testing, and treatment.

Correspondingly, research on measuring structural HIV stigma suggests the necessity to shift from attitudes or perceptions to relational structures and exclusion mechanisms themselves, thereby understanding HIV stigma as systematic screening, risk allocation, and reorganization of contact opportunities within sexual relationship structures (34), with the aim of better distinguishing the hierarchical differences between policy design, organizational implementation, and interactive consequences. Similarly, the framework for measuring homosexuality-related stigma indicates that environmental and structural variables can correspond to real dimensions of care experience, affecting medical services, communication avoidance, service accessibility, and more cautious management of identity information for sexual minorities (35). A series of studies suggests that the key to structural barriers lies not solely in whether they are perceived as discrimination and stigma, but rather in how these mechanisms are repeatedly confirmed and replicated through encoding by institutional rules, followed by organizational practices such as medical care refusal and involuntary identity disclosure, as well as interactive situations such as the avoidance of testing. Without operationalizing encoding and reproducibility, it becomes challenging to establish verifiable connections among policy texts, organizational governance, and medical interactions. Furthermore, it is impossible to clearly identify which aspects truly constitute the structural constraints that limit health initiatives for MSM in China.

Based on this, the present study defines structural inequality as a contextualized institutional combination identifiable in policy texts, organizational processes, and daily interactions. Accordingly, it employs systematic reviews and cross-analyses of policy texts and existing research findings to trace the health inequalities in HIV prevention and control faced by MSM in China by strengthening the correspondence between policy statements and behavioral consequences. This enables policy discussions to be more testable and amenable to intervention. This study proposes a coding and analysis pathway for cross-referencing Chinese HIV prevention and control policies with existing practical issues, aiming to examine the specific ways in which structural barriers are incorporated into a series of policies and assimilated into daily life, thereby changing the boundaries of action and actual life experiences. This framework clarifies the structural mechanisms that lead to the emergence of HIV prevention and control inequalities among MSM in China. Among them, policy coding aims to analyze the pathways through which structural inequalities are embedded in policy language and institutional rules and are realized through seemingly neutral terminology. This study finds that anti-discrimination legislation coexists with exclusionary civil service physical examination standards, forming a structural contradiction. This contradiction normalizes employment discrimination against HIV-positive MSM and weakens their socioeconomic foundation for accessing HIV care. Second, the intersectional stigma that accompanies HIV infection status and homosexual orientation, the contradiction between anti-discrimination legislation and the current situation of discrimination, coupled with the lack of legal recognition of same-sex relationships, leads to the refusal of care by medical institutions and the implementation of differentiated treatment. Third, the risk of involuntary identity disclosure, especially in social networks based on acquaintances in rural China, transforms HIV testing from a health-protective behavior into a perceived threat, thereby encouraging the avoidance of testing. These three paths encode structural inequality into policies and reproduce it in reality. This reproduction manifests in the repeated embodiment of exclusionary policies and medical service practices that, in reality, contradict anti-discrimination laws. This intersects with and influences individuals’ adjustments to their health actions, creating overlap and reinforcement at different levels. The findings also underscore the need to harmonize currently conflicting policies, citing successful practical cases such as Thailand’s rigorous enforcement of anti-discrimination provisions in healthcare settings and the use of digital platforms tailored for MSM to promote access to health resources. This study aims to provide empirical evidence from Chinese case studies to optimize China’s HIV/AIDS prevention and control policies, promote health equity, and safeguard the health rights and social justice of minority groups.

## 2 Method

### 2.1 Policy and literature search

#### 2.1.1 Policy search

The present study adopts a methodological framework of policy and literature analysis. Referring to Miao et al.’s framework for analyzing Chinese policy texts (36), this study will limit the included policy documents to normative documents at the central level, including the following four categories: (1) Laws promulgated by the National People’s Congress and its Standing Committee; (2) plans, action plans, and work plans issued by the State Council and its departments; (3) departmental regulations issued by departments such as the National Health Commission and the Chinese Center for Disease Control and Prevention; and (4) central normative documents and notices that have universal binding force on subordinate agencies. Local regulations, provincial implementation rules, and work documents targeting specific regions will be excluded to ensure the analysis focuses on the national institutional structure.

This study searched the following eight official policy databases and websites: National Database of Laws and Regulations, Peking University Law School’s authoritative legal database, the official website of the General Office of the State Council, the official website of the Central People’s Government of the People’s Republic of China, the official website of the Ministry of Foreign Affairs of the People’s Republic of China, the official website of the Supreme People’s Procuratorate of the People’s Republic of China, the official website of the Chinese Center for Disease Control and Prevention, and the official website of the General Office of the National Health and Family Planning Commission. The search started in March 2005, the earliest year in the current database for which the general standards for medical examinations for civil servant recruitment are available. The search ended in April 2026. The policy search was divided into two rounds, one for health and the other for employment policies. The health policy retrieval strategy used Boolean operators to combine the following keywords: “ai zi bing” OR “ai zi fang zhi” OR “xing chuan bo” OR “tong xing xing xing wei” OR “jian kang zhong guo 2030.” The employment policy search also used Boolean operators to combine the following keywords: (“gong wu yuan” OR “lu yong” OR “zhao pin” OR “jiu ye”) AND (“ti jian biao zhun” OR “ti jian tong yong biao zhun” OR “ti jian te shu biao zhun”).

#### 2.1.2 Literature search

Based on the research questions in biomedicine, public health, and health behavior, we searched the PubMed database for structural factors that affect HIV prevention and treatment in the Chinese MSM population. As an authoritative database developed by the National Library of Medicine in the United States, it systematically includes core medical, public health, and health social science journals from around the world, which can better meet the needs of this study. To cover the relevant literature as comprehensively as possible, we also conducted supplementary searches on Google Scholar. The search time range was also set from March 2005 to April 2026, in accordance with policy. The combination of search terms was: (China) AND (“men who have sex with men” OR “MSM”) AND (“HIV” OR “AIDS”) AND (“discrimination” OR “stigma” OR “employment” OR “Family support” OR “identity” OR “test” OR “testing” OR “prejudice”).

### 2.2 Inclusion and exclusion

Two researchers screened policy documents for this study, strictly adhering to the inclusion and exclusion criteria. The inclusion criteria included documents that: (1) were laws, administrative regulations, departmental rules or national plans, work plans, and normative notices at the central level of the document hierarchy; (2) clearly refer to the prevention and control of AIDS, sexually transmitted diseases, the health rights and opportunities of AIDS patients, and the relevant norms of civil servants’ employment physical examination; and (3) were released between March 2005 and April 2026. Exclusion criteria included: (1) local regulations, implementation rules, or working documents issued by provincial local governments and below; (2) purely technical operational documents that do not involve structural institutional design; (3) health notifications that only involve enterprise recruitment or are unrelated to employment standards; (4) pure news articles that do not involve structural institutional design; and (5) published before 2005 and subsequently abolished by subsequent documents.

Two researchers also screened the academic literature, strictly adhering to the inclusion and exclusion criteria. Inclusion criteria were: (1) the research object is from the Chinese mainland; (2) the research subject is the MSM population; (3) empirical research (quantitative, qualitative, mixed methods) or review research; (4) fully accessible peer-reviewed journal articles and academic publications; (5) focused on the HIV prevention, testing, and treatment behaviors of the Chinese MSM population, as well as the structural barriers and social psychological factors they face; and (6) published between March 2005 and April 2026. Exclusion criteria included studies that: (1) involved research subjects from non mainland regions of China (e.g., only Hong Kong, Macau, or Taiwan) or other countries and regions; (2) did not explicitly mention male homosexual behavior; (3) involved pure medical clinical trials that did not examine structural factors; (4) only had an abstracts available; and (5) focused on other social issues unrelated to this study.

### 2.3 Data extraction

#### 2.3.1 Policy search results

**Figure 1:**
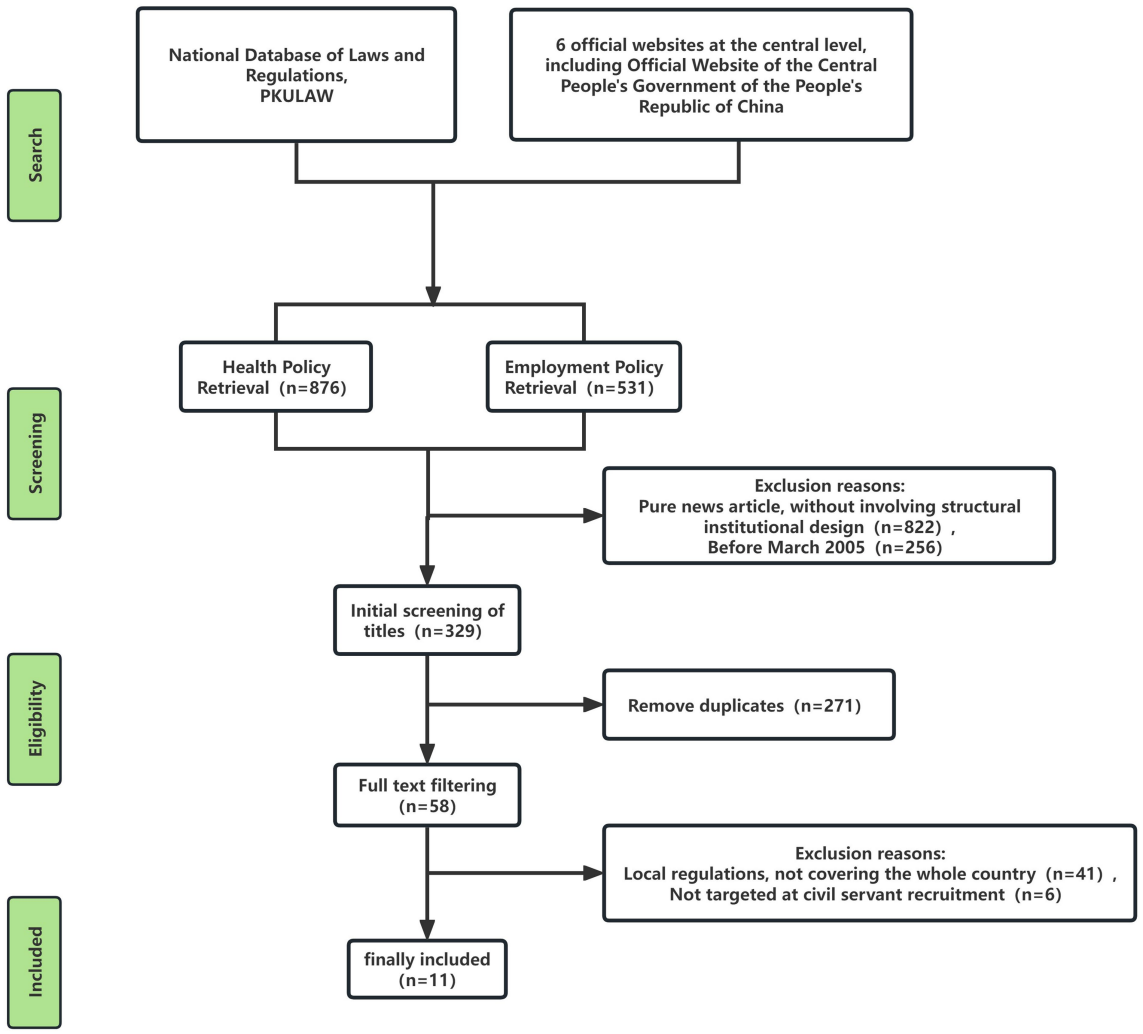
Policy identification process.

A total of 1,407 records were retrieved, including 876 for health policy and 531 for employment policy. After removing duplicate files and screening the full-text content, a total of 11 policy documents were finally included in the study, as shown in Figure 1.

#### 2.3.2 Literature search results

**Figure 2:**
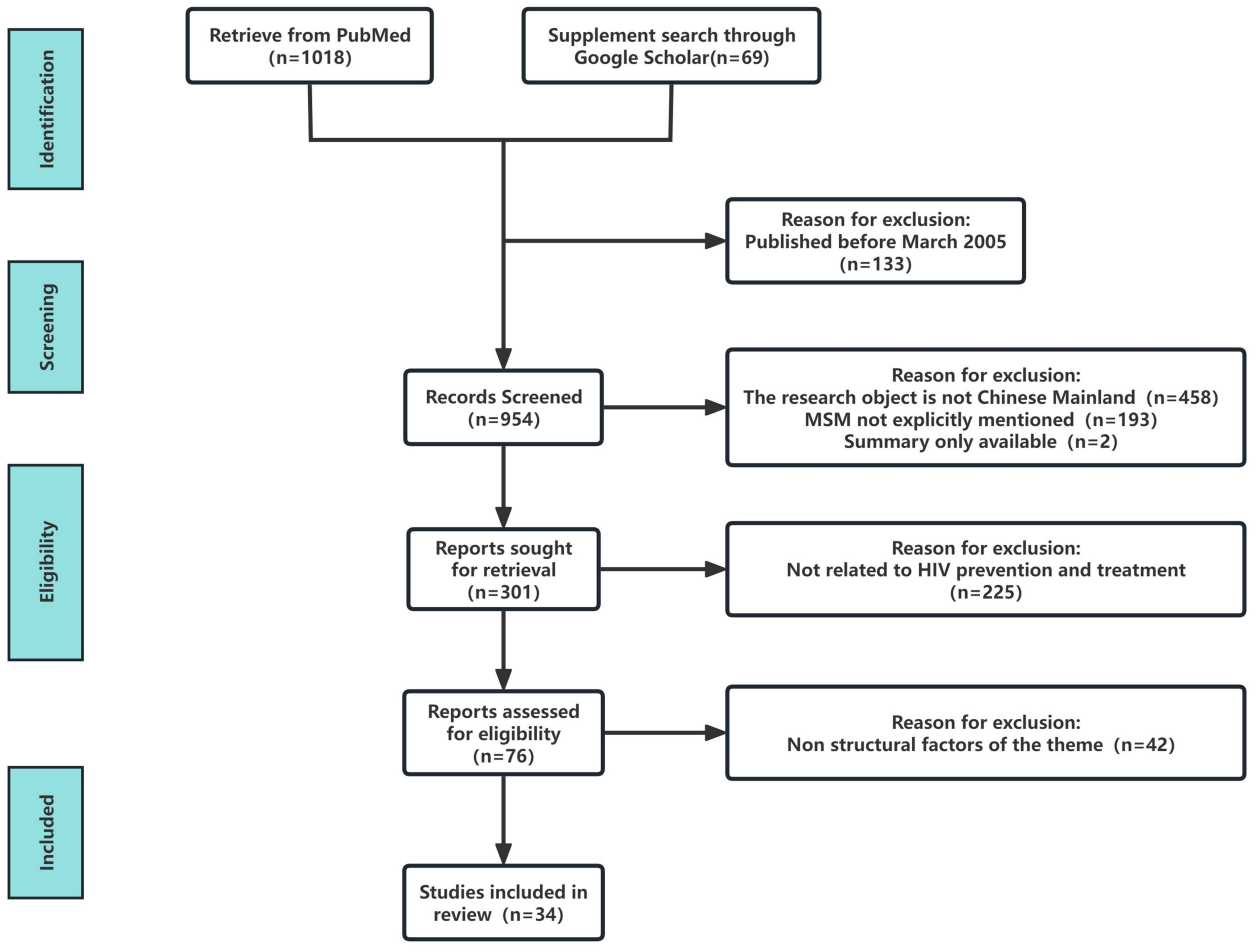
PRISMA2020 process for inclusion and exclusion of articles.

We retrieved a total of 1,087 studies, of which 1,018 were sourced from the PubMed database, and 69 were supplemented through Google Scholar search. A total of 34 items met the eligibility criteria and were included in the final analysis, as shown in Figure 2.

### 2.4 Data analysis

#### 2.4.1 Encoding the content of policy documents

After completing the policy and literature screening, the documents were imported into NVivo 12 for coding. We identified 11 initial concepts related to this study across 11 policies, and five descriptive themes (Table 1) were formed: AIDS anti-discrimination legislation, discriminatory clauses in the physical examination for civil servant recruitment, AIDS real-name system, AIDS privacy protection clauses, and other systems involving ambiguity and exclusivity.

**Table 1:** Policy document coding table.

Descriptive theme	Initial concept	Policy requirements	Source
Legislation against discrimination against AIDS	Discrimination against infected individuals is prohibited	No unit or individual shall discriminate against infectious disease patients, pathogen carriers, and suspected patients.	Law of the People’s Republic of China on the Prevention and Treatment of Infectious Diseases (revised in 2025)
		No unit or individual shall discriminate against individuals infected with AIDS virus.	AIDS Prevention and Control Regulations”(issued in 2006, revised in 2019)
		Create a non-discriminatory social environment and safeguard the legitimate rights and interests of people living with AIDS, including equal rights in medical care, employment, education, and other aspects.	China’s AIDS Prevention and Control Plan (2024-2030)
		Discrimination or illegal restrictions on employment, medical treatment, school enrollment, and other rights and interests are prohibited.	Law of the People’s Republic of China on the Prevention and Treatment of Infectious Diseases (revised in 2025)
	Guarantee employment rights	Employers shall not refuse to hire individuals on the grounds that they are carriers of infectious disease pathogens.	Employment Promotion Law of the People’s Republic of China
		No unit or individual shall discriminate against infectious disease patients, pathogen carriers, and suspected infectious disease patients.	Law of the People’s Republic of China on the Prevention and Treatment of Infectious Diseases (revised in 2025)
		No discrimination or illegal restrictions on employment, medical treatment, enrollment, and other rights and interests are allowed.	AIDS Prevention and Control Regulations (issued in 2006, revised in 2019)
	Guarantee the right to medical treatment	Medical institutions shall not evade or refuse to treat the other diseases of patients infected with HIV or AIDS.	AIDS Prevention and Control Regulations (issued in 2006, revised in 2019)
		Strengthen the responsibility system for the first consultation at medical and health institutions, and shuffling off or refusing to diagnose and treat patients is not permitted for any reason.	China’s AIDS Prevention and Control Plan (2024-2030)
Discriminatory clauses in the physical examination for civil servant recruitment	AIDS, physical examination unqualified	Gonorrhea, syphilis, chancroid, lymphogranuloma venereum, condyloma acuminatum, genital herpes, and AIDS are all unqualified.	General Standards for Physical Examination for Recruitment of Civil Servants (Trial)
		AIDS, unqualified.	Special Standards for Physical Examination for Recruitment of Civil Servants (Trial)
	Applicable to all civil servant recruitment positions nationwide	This standard applies to all civil service recruitment positions.	General Standards for Physical Examination for Recruitment of Civil Servants (Trial)
Real-name system for AIDS testing	Confirmation testing with real-name registration	The confirmation report must be completed with real-name information such as the name of the subject, ID number, current address, and contact phone number, to ensure that the sample is traceable and the data are associable.	National AIDS Testing Technical Specifications (2025 Revision)
	Positive results require an epidemic report	AIDS is a Class B infectious disease. Any unit or individual discovering a patient with an infectious disease or suspected infectious disease shall promptly report to the nearby disease prevention and control institution or medical institution.	Law of the People’s Republic of China on the Prevention and Treatment of Infectious Diseases (revised in 2025)
		It is clarified that as a Class B infectious disease, AIDS requires responsible reporting units and individuals (including medical institutions, blood collection and supply institutions, etc.) to conduct direct online reporting through the “China Disease Prevention and Control Information System” after diagnosing a positive case of AIDS. The reporting deadline is within 24 h after diagnosis.	Management Regulations for Infectious Disease Information Reporting (2015 Edition)
		Laboratories for AIDS testing and medical institutions are required to promptly report confirmed positive results of HIV antibodies to the local disease prevention and control institutions upon discovery, and cooperate with the implementation of epidemiological investigations, follow-up management, and other related tasks.	AIDS Prevention and Control Regulations (issued in 2006, revised in 2019)
		It is stipulated that AIDS testing laboratories (including screening laboratories and confirmation laboratories) must report to the local disease control and prevention institutions in accordance with the prescribed procedures after discovering positive results from HIV antibody screening or confirmation tests, to ensure that positive case information is promptly incorporated into the epidemic monitoring system.	National AIDS Testing Management Measures
HIV privacy protection clause	Voluntary testing	It is emphasized that the testing follows the principles of voluntary and informed consent, and the test results must be disclosed to the individual in a confidential manner. Only under special circumstances (such as for individuals without capacity) may the guardian be informed.	AIDS Prevention and Control Regulations (issued in 2006, revised in 2019)
		AIDS testing should adhere to the principles of voluntariness and informed consent. Before testing, it is necessary to explain the purpose, significance, and possible consequences to the testees, and obtain their written consent.	National AIDS Testing Management Measures
	Privacy protection	It is clarified that in the prevention and control of infectious diseases, personal information processing activities must protect personal privacy and must not excessively collect or use such information for non-prevention and control purposes.	Law of the People’s Republic of China on the Prevention and Treatment of Infectious Diseases (revised in 2025)
		The results of confirmatory tests should be sent in a confidential manner, and only disclosed to the individual or guardian. They should not be disclosed to third parties without consent.	National AIDS Testing Management Measures
Other systems with ambiguity and exclusivity	A minority of exclusive and vague descriptions that “are prone to spreading infectious diseases”	According to medical identification, individuals carrying the pathogen of infectious diseases shall not engage in work that is prohibited by laws, administrative regulations, and the health department of the State Council and that is prone to spreading infectious diseases, until they are cured or cleared of suspicion.	Employment Promotion Law of the People’s Republic of China
	Define marriage as the union of a man and a woman, excluding a minority of sexual orientation groups	The marriage system of monogamy is implemented.	Civil Code of the People’s Republic of China

#### 2.4.2 Encoding of literature content

The coders identified 31 initial concepts across 34 studies, ultimately forming eight descriptive themes (Table 2): employment discrimination, socio-economic status, mental health, refusal to seek medical treatment and differential care, HIV testing, sexual orientation discrimination, socio-cultural attitudes and social support, and the intervention potential of digital platforms.

**Table 2:** Literature coding table.

Descriptive theme	Initial concept	The main viewpoint of the literature	Authors
Employment discrimination	Demonstration effect of physical examination standards for civil servants	The exclusionary clauses in the physical examination standards for civil servants will have a demonstration effect in the job market.	Burki TK (58)
	Legal protection and practical challenges coexist	Anti-discrimination legislation and practical challenges coexist.	Duan & Xie (14), Murzakaev (50), Tsang E. Y. (51)
	The impact of employment exclusion	Employment exclusion harms the socioeconomic status and mental health of MSM.	Burki TK (58), Duan & Xie (14), Hu et al. (43), Wang et al. (20), Zhao et al. (41), Chen et al. (42)
Socioeconomic status	The moderating effect of socioeconomic status	Demographic characteristics such as socioeconomic status are significantly correlated with HIV prevention behaviors.	Zhang et al. (13), Chen et al. (19), Chen et al. (42), Ni et al. (24), Huon et al. (55)
		Low socioeconomic status moderates the extent of discrimination.	Duan & Xie (14), Chen et al. (42), Tsang E. Y. (51)
	Employment situation and income	Occupational status serves as a crucial measure.	Duan & Xie (14), Tsang E. Y. (51)
	Willingness to pay	MSM with low socioeconomic status exhibit lower willingness to pay for HIV-related services.	Cui et al. (5), Zhang et al. (13), Chen et al. (19), Chen et al. (42), Huon et al. (55)
	Health literacy	The significant differences in access to medical resources between urban and rural areas have a notable impact on health literacy.	Chen et al. (19), Ni et al. (24)
		Rural MSM are at a disadvantage in terms of their knowledge of HIV.	Chen et al. (19), Ni et al. (24)
Mental health	Employment pressure	Institutional employment exclusion affects stress status.	Burki TK (58)
	Moral anxiety	Feel pressured by the “improper” moral standards.	Zhang et al. (13), Steward et al. (15), Liang & Huang (23), Smith et al. (48), Tsang E. Y. (51)
		Due to the moral pressure felt by a minority due to their sexual orientation.	Gong & Miao (16), Steward et al. (15), Pyun et al. (22), Zhao et al. (41)
	Depression	HIV positivity has an impact on depression.	Wang et al. (20), Wu et al. (21), Liang & Huang (23), Yang et al. (46)
		Sexual orientation has an impact on depression.	Gong & Miao (16), Wu et al. (21), Pyun et al. (22), Liang & Huang (23), Yang et al. (46)
	Health anxiety	Fear of being infected with HIV.	Wang et al. (20), Wu et al. (21), Liang & Huang (23), Hu et al. (43)
	Discrimination fear	Fear of social discrimination brought by HIV-positive status.	Wang et al. (20), Zhao et al. (41)
		Fear of employment discrimination due to being HIV-positive and being identified as an MSM.	Chen et al. (19), Wu et al. (21), Pyun et al. (22), Liang & Huang (23), Zhao et al. (41), Yang et al. (46), Tsang E. Y. (51)
		Fear of medical discrimination due to being HIV-positive.	Wang et al. (20), Hu et al. (44), Yang et al. (46), Smith et al. (48), Tsang E. Y. (51), Dong et al. (57)
	Minor pressure	The pressure of internalized homophobia and self-stigma.	Pyun et al. (22), Liang & Huang (23), Yang et al. (46)
		I feel pressured due to the lack of policy support.	Zhang et al. (13), Murzakaev (50), Tsang E. Y. (51)
		“Believing that homosexual behavior is wrong” and feeling pressured due to a lack of social support.	Xie & Peng (18), Zhao et al. (41), Tsang E. Y. (51)
		Feeling stressed due to lack of family support.	Steward et al. (15), Wang et al. (20), Pyun et al. (22), Zhao et al. (41)
Denial of diagnosis and differentiated care	Refusal of treatment	PLWH encounter refusal or prevarication in outpatient and inpatient services.	Jia et al. (17)
		The behavior of refusing diagnosis exists among medical staff.	Jia et al. (17), Hu et al. (44), Xu et al. (49)
	Wound management concerns	Medical staff have concerns about wound management.	Hu et al. (44)
	Implement differentiated care	The quality of care received by HIV-positive MSM is significantly lower than that of other patients. HIV antibody testing is conducted without patient consent.	Smith et al. (48), Dong et al. (57)
	Biased medical treatment	Doctors’ prejudice against sexual minorities undermines the accessibility of medical services, and older doctors tend to hold stronger prejudices.	Xu et al. (49), Dong et al. (57)
HIV test	HIV testing rate	The sample detection rate is relatively high and the test is primarily targeted at the MSM population.	He et al. (54), Yang & Sun (52)
		The detection rate for MSM in rural areas is significantly lower.	Chen et al. (19), Ni et al. (24)
		30% tested after high-risk sexual behavior.	Zhou et al. (53)
	Uneven distribution of medical resources between urban and rural areas	The allocation of medical and health resources to urban and rural areas is uneven, leading to a greater reliance on informal channels for rural MSM to obtain treatment medications.	Chen et al. (19), Ni et al. (24)
	Limited access to information channels	It is difficult for MSM to obtain health education resources related to their identity through mainstream channels.	Miao & Chan (59), Zhang et al. (13)
	HIV testing coverage	HIV testing in China is primarily targeted at men who have sex with men (MSM).	Dou et al. (4)
		The detection coverage is insufficient.	Dou et al. (4), Cui et al. (5), Zhang et al. (13), Tucker et al. (45), Zhou et al. (53), Dai et al. (60)
	Risk of disclosing positive results	Involuntary disclosure of identity poses risks.	Wang et al. (20), Wu et al. (21), Pyun et al. (22), Hu et al. (43)
		The involuntary disclosure of MSM in rural areas leads to the amplification of risks.	Chen et al. (19)
Sexual orientation discrimination	Traditional cultural concepts	Under traditional Chinese cultural concepts, homosexuality is subject to ethical criticism.	Steward et al. (15), Pyun et al. (22), Tsang E. Y. (51)
	Medical field	Discrimination against sexual minorities by doctors leads to differential treatment in medical care.	Smith et al. (48), Xu et al. (49), Dong et al. (57)
		Some experts believe that being MSM implies a potential risk of HIV infection.	Murzakaev (50)
	Discrimination by officials and their staff	Government officials discriminate against homosexuals.	Tsang E. Y. (51), Burki TK (58)
Sociocultural concepts and social support	Traditional cultural concepts and Confucian filial piety	The filial piety concept of marriage and procreation constitutes a unique cultural pressure source for Chinese MSM.	Gong & Miao (16), Steward et al. (15), Pyun et al. (22)
	MSM identity and social support	MSM identity negatively affects the social support they receive.	Zhang et al. (13), Xie & Peng (18), Pyun et al. (22), Zhao et al. (41), Tsang E. Y. (51)
	HIV positivity and social support	HIV positivity can weaken the acquisition of social support.	Wang et al. (20), Zhao et al. (41), Murzakaev (50)
The intervention potential of digital platforms	Prevention and intervention on digital platforms such as Blued	Through collaboration with political forces, Blued has evolved into a health education platform.	Miao & Chan (59)
		Providing intervention materials through a platform is an economical and effective strategy.	Dai et al. (60), Huang et al. (61)
	Anonymous identity	The platform creates social affordances for identity anonymity.	Miao & Chan (62)
	Sense of community belonging	The platform creates social affordances for community belonging.	Miao & Chan (62)

#### 2.4.3 Topic analysis

After coding the policy documents and academic literature, we conducted a cross-analysis of the two data sources to integrate them, ultimately identifying three analytical themes (Table 3): discriminatory employment exclusion and mental health, unequal medical treatment under structural stigma, and inadequate and avoided medical testing. Based on the determinants of health in society, health inequality is believed to be rooted in individuals’ social status, power relations, and resource allocation. These three themes form interactive relationships at the macro environment, healthcare industry operations, and individual behavior levels, forming the three major factors of health inequality influencing HIV prevention and treatment among the Chinese MSM population. Discriminatory employment exclusion and mental health constitute an unequal environment and individual psychological foundation, while the contradiction between non-discriminatory policies and actual discriminatory practices constitutes medical injustice. Both factors, along with the risk of involuntary disclosure, contribute to individuals’ avoidance of testing and, in turn, reinforce the structure and stigma.

**Table 3:** Cross analysis table.

Analytical theme	Descriptive themes incorporated into policy formation	Descriptive themes formed by incorporating literature
Discriminatory employment exclusion and mental health	Discriminatory clauses in the physical examination for civil servant recruitment	Employment discrimination
	Legislation against discrimination against AIDS	
	Other systems with ambiguity and exclusivity	
		Socioeconomic status
	Discriminatory clauses in the physical examination for civil servant recruitment	Mental health
	Other systems with ambiguity and exclusivity	Sexual orientation discrimination
	Legislation against discrimination against AIDS	Sociocultural concepts and social support
	Discriminatory clauses in the physical examination for civil servant recruitment	
Inequitable medical treatment under structural stigma	Real-name system for AIDS testing	Denial of diagnosis and differentiated care
	HIV privacy protection clause	
	Other systems with ambiguity and exclusivity	Sexual orientation discrimination
	Legislation against discrimination against AIDS	Sociocultural concepts and social support
	Discriminatory clauses in the physical examination for civil servant recruitment	
Inadequacy and avoidance of medical testing	Real-name system for AIDS testing	HIV test
	HIV privacy protection clause	
	Other systems with ambiguity and exclusivity	Sexual orientation discrimination
	Legislation against discrimination against AIDS	Sociocultural concepts and social support
	Discriminatory clauses in the physical examination for civil servant recruitment	
	Real-name system for AIDS testing	The intervention potential of digital platforms
	HIV privacy protection clause	

## 3 Results

### 3.1 Discriminatory employment exclusion and mental health

Health discrimination in employment refers to the behavior of labor organizations and employers that unreasonably harms workers or deprives them of their rights to equal opportunities and treatment in employment on the grounds of their health status. The Chinese government has consistently made sustained efforts to eliminate AIDS-related employment discrimination. Article 3 of the Regulations on the Prevention and Control of AIDS (Decree No. 457 of the State Council), promulgated by the State Council in 2006, clearly stipulates that no unit or individual may discriminate against people infected with the AIDS virus or patients with AIDS and their families, and that their legitimate rights and interests such as employment and medical treatment are protected by law (37). When revised in 2019, this clause was completely retained and has been used thus far (38) with a view to eliminating social discrimination against AIDS patients in the employment context. However, institutional tension remains between the aforementioned anti-discrimination legislation and the current multi-position employment medical examination and recruitment standards. In the revised Employment Promotion Law of the People’s Republic of China in 2015, Article 13 clearly stipulates that carriers of infectious diseases identified through medical examination shall not engage in work that is prohibited by laws, administrative regulations, and regulations of the State Council’s health department and which is likely to cause the spread of infectious diseases before being cured or cleared of suspicion (39). The formulation of this regulation has positive significance for controlling the spread of HIV/AIDS, but the vague expression of “easy spread of infectious diseases” in the regulation leaves a lot of room for interpretation in practice, often leading to greater feasibility of employment exclusion in practical operations. In 2005, the Ministry of Personnel and the Ministry of Health issued the General Standards for the Physical Examination of Civil Servants (for Trial Implementation). Article 18 clearly lists AIDS as an unqualified item in the physical examination (40). This exclusionary clause has not yet been abolished and continues to apply to the recruitment of civil servants and public institution staff at all levels throughout the country. Under China’s political and administrative system, the medical examination standards for civil servant recruitment are highly centralized and unified, and local medical examination standards are strictly implemented in accordance with central standards. Xie and Duan summarize this phenomenon as the coexistence of legal protection and practical challenges (14). Although the national legislation aims to prevent discrimination against people infected with the AIDS virus, these efforts are often hindered by other laws and obvious and illegal discrimination. The growing concern and stigma within Chinese society about the spread of AIDS mean that a large proportion of AIDS patients are still discriminated against and unable to work or lose their jobs.

The dual identity of MSM with HIV/AIDS means that they face greater maladjustment under discriminatory employment policies. The representative position of civil servants’ employment in China’s labor market demonstrates the exemplary effect of discriminatory employment provisions (14). The exclusion of homosexual identity by state discourse also leads to more discrimination against this group by government officials (41), which leads to widespread health and sexual orientation discrimination in the current employment market, creating employment inequality. The HIV prevalence rate among MSM is significantly higher than the national average (42). Under the mass media reporting framework, the public has gradually realized that MSM are more susceptible to HIV infection, leading to greater prejudice against gay men than against lesbian women (41). Once the dual identity of MSM living with HIV is exposed, it becomes difficult for them to secure a stable job.

Work and employment shape population health, and occupational status has become a key demographic indicator of health-risk behaviors among MSM in the current socio-economic environment. Exclusive employment policies with a demonstration effect can exacerbate the burden of HIV/AIDS prevention and mental health risks by undermining the socio-economic status of MSM (14). Chen and Chai pointed out that demographic characteristics such as socioeconomic status are significantly correlated with HIV prevention behaviors (19). Due to the lack of stable economic security and institutionalized social protection of MSM who are economically vulnerable and afflicted with HIV, they exhibit lower willingness to pay for and greater avoidance of HIV-related services (14). Unemployment or insecure employment restricts access to prevention resources such as PrEP and hinders treatment adherence among HIV-infected MSM, and those in unstable work often also exhibit weaker perceptions of condom social norms and more negative protective attitudes (43). This is because individuals who experience employment discrimination and social isolation often lack the necessary social capital compared to those with strong social support, which in turn leads to a more inaccurate assessment of their own infection risk and clinical support eligibility (24).

Institutional employment exclusion can also harm the health of MSM through psychological pathways. In the Chinese cultural interpretation of responsibility, traditional gender role norms construct men as the core pillar of the family economy. Society and family have high and rigid expectations for men’s employment quality and career achievements. MSM who are institutionally excluded from employment bear higher economic marginalization pressure and moral anxiety. The increase in negative emotions significantly predicts the occurrence of depressive symptoms in HIV-positive MSM (41). Meanwhile, most Chinese people hold a conservative attitude towards homosexuality in general, believing that homosexual behavior is wrong and often associating it with promiscuous lifestyles and the stigma of AIDS (18); even MSM who have not disclosed their HIV-positive status face employment discrimination. The unfavorable employment atmosphere created by discriminatory employment regulations poses significant occupational risks to MSM. To avoid potential professional hazards, most MSM conceal their identities in the workplace. This long-term, active self-imposed isolation requires a continuous investment of cognitive and emotional resources to monitor one’s own behavior, manage social cues, and suppress true expressions. It also significantly depletes psychological coping resources, thereby inducing mental health issues such as depression and suicide (42).

### 3.2 Inequitable medical treatment under structural stigma

In China’s macro-level AIDS response, stigma towards MSM arises from structural forces resulting from the combined effects of social structures and cultural paradigms. Unlike the minority stress model in Western contexts, the pressure borne by Chinese MSM is influenced by family orientation and heterosexual hegemonic culture. In a cultural context of mainstream rejection, stigmatization towards sexual minorities often manifests as multidimensional minority stress due to a lack of policy, family, and social support (13, 24), creating culturally entrenched identity rejection in healthcare settings.

HIV-1 is the predominant viral type in the global AIDS epidemic, and the HIV-1 subtype predominantly circulating in China is HIV-1 CRF_01AE, which is also the most prevalent subtype among MSM populations. While the Chinese government has formulated multiple documents such as the “Regulations on the Prevention and Control of AIDS” and the “13th Five-Year Action Plan for the Control and Prevention of AIDS in China” to eliminate AIDS discrimination and protect the medical rights of infected individuals, due to the risk of infection during medical procedures caused by blood transmission, many AIDS patients are denied treatment for diseases not related to HIV (DNRH) (44). Although anti-discrimination policies have elicited a certain degree of response at the social cognitive level, there remains a gap in their actual implementation within medical institutions. Multiple studies have shown that people living with HIV (PLWH) face a high proportion of denial of diagnosis, with approximately 42.2% of infected outpatients and 63.0% of infected inpatients encountering denial of diagnosis or prevarication during their most recent medical visit (44). Jia et al. investigated the Huaxi region, where HIV is more prevalent and MSM are more concentrated. The results showed that 65.1% of medical staff had concerns about wound management (17), and fear may lead to differential treatment of infected individuals, especially MSM. Surveys conducted in the economically developed, highly educated eastern coastal areas also revealed that the proportion of healthcare workers who refused to seek medical treatment reached 38.6% (45). In the absence of a comprehensive compensation mechanism for occupational exposure and safety and protection guarantees, medical institutions tend to adopt defensive medical strategies, pushing high-risk groups to specialized infectious disease hospitals by setting invisible thresholds. When individuals no longer believe they are capable of obtaining fair treatment or effectively controlling their condition within the complex medical system, seeking medical care may be perceived as a potentially humiliating experience rather than as a path to recovery, leading to a reduced willingness to seek treatment or to a greater likelihood of seeking informal medical channels, further increasing the risk of infection transmission (44, 45).

Compared to other infected individuals, the prominent structural stigma faced by HIV-positive MSM is the intersectional stigma of being both PLWH and MSM. Research indicates that there is a significant interaction between HIV-related stigma and stigma towards sexual minorities (46). The combination of these identities has a more profound impact on an individual’s depression, anxiety, quality of life, and psychological resilience than stigma towards either identity alone. On the one hand, as an important component of the social structure, the Civil Code strictly defines marriage as the union of a man and a woman (47), thereby universalizing the recognition of the gendered family structure. On the other hand, as the birthplace of Confucian culture, China’s sexual culture is founded on traditional ethical roots. Concepts advocated by Confucianism, such as “there are three forms of disobedience, but the greatest is to have no offspring,” enshrine heterosexual marriage and childbearing as the core of filial piety and family continuation. Meanwhile, homosexual behavior is often relegated to the fringes of ethical order or even criticized, laying the social groundwork for discriminatory attitudes. The resulting structural intersectional stigma has a significant negative impact on HIV-positive MSM seeking medical support. Research by Smith et al. found that HIV-positive MSM receive significantly poorer quality of care than other patient groups, and training interventions aimed at reducing stigma have the least effect on improving the quality of care for this population (48). This structural stigma is particularly prominent in the field of surgery. Although there is a significant positive correlation between a doctor’s knowledge level regarding HIV and their actual willingness to treat patients, existing prejudices against sexual minorities can still significantly undermine the accessibility of medical services (49). Furthermore, older doctors tend to hold greater prejudice against MSM and a lower willingness to diagnose and treat them (49), leading to higher thresholds among MSM patients for complex surgeries such as liver transplants. Hu et al.’s research corroborates this (44), as men who have not disclosed their MSM or HIV infection status are less likely to be denied treatment. Therefore, to obtain equal access to medical care, HIV-positive MSM often take risks by concealing their medical history. Compared to countries like Thailand, which actively reduce discrimination in medical settings through legal and regulatory measures, a significant gap remains in China in establishing legal protections against stigmatization for specific groups (50).

The stigmatization process in the medical field is further complicated by the institutional differences inherent in the urban–rural system. When a large number of MSM workers migrate from rural areas to cities, their status as rural immigrants intersects with their sexual minority identity and HIV infection status, resulting in multiple stigmatizations (15). Meanwhile, due to the imbalance in the allocation of medical and health resources between urban and rural areas, sexual minorities in regions lacking specialized medical services rely more on informal channels to obtain therapeutic drugs, increasing risks associated with medication safety. Tsang analyzed the marginalized situation of homosexual sex workers in China’s medical system from the perspective of “biopolitics” (51). Due to the government’s non-recognition of their sexual orientation, the MSM community struggles to effectively access urban medical resources, and their right to medical care is suppressed under the dual pressures of social discrimination and administrative supervision. The urban–rural disparities in medical resources also significantly affect health literacy (43). Compared to urban MSM, individuals in rural areas, despite having a weaker social support network and thus holding a higher subjective vigilance towards infection risks, still lag behind in terms of actual HIV knowledge acquisition, diversity of information acquisition channels, and high-frequency testing behavior (19), exhibiting a contradiction of high risk perception and low protective behavior.

### 3.3 Inadequacy and avoidance of medical testing

The findings on HIV testing status among MSM in China present structural tension. While large-scale HIV testing in China is primarily targeted at gay men (52), the research conducted by Zhuang (5) and Chen et al. (19) showed that the testing coverage rate remains insufficient, especially among rural MSM, where it is significantly lower (19). Zhou et al. also found that only 30% of MSM chose to get tested after engaging in high-risk sexual behavior (53). Conversely, a cross-sectional study conducted on the internet and involving 7,629 MSM found that 87.1% of the respondents had undergone HIV testing (54). Another study also suggests that the HIV testing rate among MSM in China is significantly higher than 79% (55). The differences in these data reveal the structural differentiation in testing behavior among MSM in China. Respondents recruited through the internet tend to have higher digital literacy, stronger health awareness, and more connections within the MSM community, and their testing rate is significantly higher than the national average (54). However, in marginalized sub-groups lacking community connections and digital accessibility, the problem of insufficient testing is more pronounced (19). For MSM who are older, of lower socioeconomic status, or rural and are in a “deep closet” state, insufficient testing and avoidance remain major obstacles to HIV prevention and control. The reasons for this include various structural factors such as the real-name testing system, identity disclosure risks, and rural social networks in rural society. We therefore believe that although testing coverage has improved among some populations, testing avoidance, as a behavioral pattern driven by structural factors, still persists in the broader Chinese MSM population and has a greater impact on HIV prevention and control among marginalized subpopulations.

Testing is the foundation of AIDS prevention and control efforts. HIV-infected individuals often exhibit no specific symptoms and signs during the pre-AIDS stage, and it is necessary to detect possible infection through screening and confirm the diagnosis through supplementary testing before initiating antiviral therapy. During treatment, regular viral load testing is required to assess efficacy and monitor for drug resistance in a timely manner. At the same time, including CD4 + T lymphocyte testing to evaluate immune reconstitution status is essential for effectively achieving the goal of antiviral therapy. Currently, commercial HIV infection tests widely used in China are predominantly Ab tests or Ag/Ab tests. Ab tests primarily detect specific antibodies produced by the human body after HIV infection; these antibodies are usually detectable 3–12 weeks following infection, with a relatively long window. Ag/Ab tests can simultaneously detect HIV p24 antigen and antibodies, allowing detection of infection around 2–4 weeks after exposure, with a shorter window and higher sensitivity. The World Health Organization recommends HIV self-testing (HIVST) as an extension of routine HIV testing services. HIVST kits are typically based on antigen–antibody reactions, detecting HIV antibodies or both antigen and antibody in the sample to determine whether an individual is infected with HIV. Although the use of HIVST is gradually expanding in China, the adoption rate among the MSM population remains relatively low (5), and the development of HIVST strategies in China still faces challenges.

China’s current HIV testing management system implements real-name registration for confirmatory testing. According to the requirements of the “National Technical Specifications for HIV Testing (2025 Revision),” the testee’s true identity must be recorded for HIV confirmatory testing, and positive results must be reported to the local Center for Disease Control and Prevention in accordance with the law (56). From the perspective of public health management, the design of the real-name system aims to ensure effective tracking, treatment referral, and epidemic monitoring of individuals with positive test results, and its public health objectives are clearly reasonable. However, the system’s value-neutral design constitutes a form of structural violence in practice. Although the system design itself lacks discriminatory intent, it can conceal harm to marginalized groups through its equal application. For groups under minority pressure, involuntary disclosure not only subjects individuals to the health consequences of HIV infection but also to further discrimination resulting from the disclosure of their sexual orientation as homosexual.

Among these factors, the structural stigma risk created by involuntary identity disclosure and the psychological mechanism of self-stigma mutually reinforce MSMs’ avoidance of testing. Some public health experts believe that being gay is equivalent to being at risk of contracting AIDS (52). Multiple studies have also shown that a large number of infected individuals are subjected to HIV antibody testing (57) and disclosure of their HIV status (58) without their consent. Although the “AIDS Prevention and Control Regulations” clearly stipulate that the privacy of infected individuals is protected by law, in practice, infected individuals may still face involuntary disclosure when working or seeking medical care in public institutions (58). Under the long-term influence of traditional values, the widespread stigma against sexual minorities and people with HIV/AIDS in Chinese society mediates the life experiences and happiness perception of HIV-positive MSM (42), leading to extreme fear of HIV infection among MSM with high self-stigma. They also anticipate the social discrimination and rejection that may arise from positive test results. Under these dual anxieties, individuals often perceive testing behavior as risking identity exposure rather than as a health-protection measure and therefore avoid testing. This situation is more pronounced in rural China, an acquaintance-based society structured around clan networks and characterized by prominent local features. A person’s social existence is not perceived as an atomized individual but is deeply embedded in family lineage and kinship networks. In rural areas with a strong clan culture, family reputation is a more important form of social capital, and individual behavior reflects not only the self but also visible parents, siblings, and even the entire family. Consequently, rural MSM are more cautious in their risk assessment when it comes to HIV testing (19). The involuntary disclosure of test results may stigmatize the family, causing shame and leading to individual exclusion within the family, which serves as the foundation of their social interaction. This, in turn, may result in further marginalization in rural public life.

Furthermore, based on the social ideological environment centered around Confucian culture, the mere mention of sex often evokes a negative reaction; this has kept sex education on the fringes of Chinese educational content. Sexual topics have long been regarded as private issues that should not be publicly discussed in Chinese society. This cultural habit has affected the visibility of sexually transmitted disease (STD) testing tools in the public sphere. At the same time, the state’s control over media content involving homosexuality has further narrowed the formal channels through which MSM groups can access targeted health information. An ethnographic study (59) notes that, despite Xinhua News Agency’s positive coverage of the gay website Danlan.org during the 2008 Beijing Olympics to showcase Beijing’s open image, overall, LGBTQ-related content still faces strict censorship in China. Traditional media face restrictions in reporting on LGBTQ+ issues, and social media platforms must also comply with the Cyberspace Administration of China’s content management requirements. MSM therefore struggle to obtain culturally relevant health education resources related to their identity through mainstream channels, as well as to access channels for active testing and intervention.

## 4 Discussion and suggestions

To address the limited effectiveness of content dissemination through mainstream channels, numerous studies have emphasized the potential of digital media platforms (5, 60–62), particularly the constructive role of local same-sex social platforms, as alternative channels (59). Danlan.org, operated by Blue City Holdings Ltd. and later developed into “Blued,” is a dating website specifically designed for sexual minorities. It is headquartered in Beijing and is also the world’s largest gay dating app. The platform actively seeks cooperation with the Chinese Center for Disease Control and Prevention to extend its positive role as an MSM health education platform. In previous practice, Blued tested over 10,000 MSM for HIV at its Beijing headquarters within 1 year (59). An exploratory real-world study further discovered the effectiveness of providing HIV testing intervention materials to MSM through Blued and concluded that such digital platforms are a cost-effective strategy for increasing HIV testing rates and promoting health interventions among MSM (60). In subsequent research from an affordance-theory perspective, Miao et al. concluded that platforms such as Blued not only provide the technical affordance of geolocation but also create social affordances, including identity anonymity, community belonging, and access to health information (62). This has shaped the potential of such digital media platforms.

On February 27, 2026, Geng Le, the founder of Blue City Holdings Ltd., launched a new app named “Qing,” which ranked fifth in the social networking category on Apple’s App Store on its first day of release. It is evident that the demand for social platforms among MSM has not diminished. Social media, as a digital haven, remains vital in connecting the MSM community, providing a social space and emotional support for MSM who have long been subjected to social stigma and psychological oppression. In China’s current cultural and political context, traditional media may struggle to effectively serve as an infrastructure for the healthy development of the MSM community in the short term. Gay social media platforms represented by Blued and Qing may be able to achieve broader population coverage at lower costs, unlocking their potential as channels for AIDS prevention and control interventions. Integrating prevention and control information into existing social applications is not only feasible but also well-received by users (61). In the future, those designing HIV/AIDS prevention and control measures for MSM groups should consider collaborating with social media platforms for same-sex interactions. Through such platforms, prevention and control knowledge can be disseminated, HIVST links can be distributed, and feedback channels for issues such as unequal access to medical care and employment can be created, thereby compensating for the deficiencies of traditional offline medical testing and intervention. Additionally, in terms of design, attention should be paid to leveraging the rationality of same-sex digital media platforms, avoiding the excessive embedding of disease-related content that may elicit user resentment, and circumventing the issue that Blued once faced, where the overemphasis on HIV prevention and control information was perceived by users as reinforcing stigmatization against the gay community (59). Future research should focus more on the effectiveness of localized Chinese social media platforms for same-sex interactions in HIV prevention and health equity among MSM. Through empirical quantitative, qualitative, or mixed-methods research, support should be provided to develop practical and effective prevention and treatment models that promote the healthy development of the MSM population.

The key to achieving AIDS prevention and control among MSM in China lies in systematically addressing the inequalities in the social structure, based on increasingly mature clinical and digital intervention methods. Although China has achieved phased results in advancing the prevention and control goals of the Joint United Nations Program on HIV/AIDS, structural barriers still hinder effective coverage of all aspects of diagnosis and treatment. The future intervention path should shift from focusing on single biomedical cures to multi-dimensional coordination based on social networks, thereby constructing a comprehensive support system that incorporates individual active testing, family support, community cooperation, and macro-policy integration. At the same time, future policy formulation and research should focus on destigmatizing and standardizing diagnostic and treatment services and improving the accessibility of prevention methods such as PrEP. This not only requires promoting clinical technology but also relies on top-level policy design to eliminate social discrimination, ensure employment as the foundation of health security, enhance individuals’ initiative and access to medical testing, and guarantee health equity for MSM.
